# Objective classification of zonular weakness based on lens movement at the start of capsulorhexis

**DOI:** 10.1371/journal.pone.0176169

**Published:** 2017-04-20

**Authors:** Saori Yaguchi, Shigeo Yaguchi, Yukari Yagi-Yaguchi, Tadahiko Kozawa, Hiroko Bissen-Miyajima

**Affiliations:** 1 Department of Ophthalmology, Tokyo Dental College Suidobashi Hospital, Tokyo, Japan; 2 Kozawa Eye Hospital and Diabetes Center, Mito, Japan; 3 Department of Ophthalmology, Tokyo Dental College Ichikawa General Hospital, Chiba, Japan; Rush University Medical Center, UNITED STATES

## Abstract

**Purpose:**

To quantify zonular weakness based on lens movement at the start of continuous curvilinear capsulorhexis (CCC) and establish a classification system for it.

**Setting:**

Kozawa Eye Hospital and Diabetes Center, Mito, Japan.

**Design:**

Retrospective interventional case series.

**Methods:**

We examined 402 consecutive eyes of 316 patients who underwent CCC, phacoemulsification and aspiration (PEA), and intraocular lens (IOL) implantation. The movement of the lens capsule was measured using images from video recordings of the CCC procedure. Zonular weakness was classified based on the shifted distance: Grade I, less than 0.20 mm; Grade II, 0.20–0.39 mm; and Grade III, greater than 0.40 mm. For each of these grades, we examined the use of the capsule stabilization device during PEA, the surgical procedure for lens removal, and IOL fixation.

**Results:**

We classified 276 eyes (68.6%) as Grade I, 102 eyes (25.4%) as Grade II, and 24 eyes (6.0%) as Grade III. As the grade increased, the use of the capsule stabilization device in PEA and scleral suture fixation of IOL increased.

**Conclusions:**

Zonular weakness was quantified by measuring the movement of the lens capsule. An objective classification of zonular weakness based on lens movement may be useful for selecting the appropriate device and procedure during cataract surgery.

## Introduction

Standard cataract surgery using phacoemulsification and aspiration (PEA) can be safely performed provided that the zonule of Zinn is healthy and appropriately supports the lens; following the surgery, the in-the-bag intraocular lens (IOL) fixation can be performed. However, in cases where there is zonular instability, the rate of intraoperative complications increases, with a risk of posterior capsule rupture, nucleus drop, or vitreous prolapse. The use of capsule stabilization devices and modification of surgical techniques enable surgeons to perform successful PEA with endocapsular posterior chamber IOL fixation. Appropriate use of an adjunct capsule stabilization device can improve the safety and outcome of procedures performed in patients with zonular instability. Hara et al [1] introduced the concept of the equator ring, which is the prototype for the capsular tension ring (CTR). Cionni and Osher [2] introduced a modified CTR for use in patients with significant zonular compromise or lens subluxation; subsequently, several types of endocapsular support devices to manage lens subluxation have been reported [3–5]. Cases of severe zonular compromise require intracapsular cataract extraction (ICCE), pars plana vitrectomy (PPV), or scleral suture fixation of IOL.

It is crucial during cataract surgery to properly ascertain the condition of the zonule because this is a determining factor pertaining to whether the surgery can be safely conducted. Although preoperative findings of lens subluxation, zonular dialysis, or phacodonesis are direct signs of severe zonular weakness, it is difficult to identify minor and moderate zonular weakness. Zonular strength is best assessed intraoperatively through the use of several maneuvers and observations. Findings at the initiation and during continuous curvilinear capsulorhexis (CCC), such as movement of the entire capsular bag during propagation of the capsular flap and anterior capsule striae, are considered to be the intraoperative litmus tests for zonular instability [6]. Shingleton et al reported the retrospective case review of pseudoexfoliation syndrome (PXS) eyes with preoperative and intraoperative signs of zonular weakness, PXS eyes and non-PXS eyes without zonular weakness undergoing PEA and IOL implantation, and concluded that postoperative complication rates are higher in PXS eyes with preoperative and/or intraoperative signs of zonular weakness [7]. In a previous experimental study using a model of weak zonules in an extracted porcine eye, we demonstrated that the shifted distance of the lens capsule at the start of CCC was correlated with the degree of zonular dehiscence, validating the use of the measurement of lens movement to determine the degree of zonular weakness [8]. If the lens movement at the start of CCC can be measured in a clinical study using the same methods as we used in the experimental model of weak zonules, we believe that zonular weakness can be objectively classified based on the degree of this lens movement.

In the present study, we quantified lens movement at the start of CCC in eyes that underwent PEA and IOL implantation. We then classified zonular weakness into three groups based on the shifted distance of the lens. We also investigated the surgical procedures used for each of these groups.

## Materials and methods

### Patients

This study included 402 eyes of 316 consecutive patients who underwent planned PEA and IOL implantation between October 2014 and September 2015 at the Kozawa Eye Hospital and Diabetes Center. Severe zonular deficiency such as severe phacodonesis, lens subluxation, lens luxation into the anterior chamber, and dropped nucleus into the vitreous cavity that could be detected preoperatively were excluded from this study because CCC could not be performed and the planned ICCE and PPV were performed instead. ICCE was performed on eyes with hypermature cataract, and these eyes were also excluded. Intumescent cataract was also excluded because CCC was performed using a microforceps after staining with trypan blue. The mean age of the patients was 74.0 ± 12.9 years (range, 18–100 years). Nuclear sclerosis based on the Emery-Little classification were grade 1 (15 eyes, 3.73%), grade 2 (102 eyes, 25.37%), grade 3 (248 eyes, 61.69%), grade 4 (36 eyes, 8.96%), and grade 5 (1 eye, 0.25%). Predisposing factors for weak zonules were PXS (58 eyes, 14.43%), a history of laser iridotomy (23 eyes, 5.72%), a history of blunt trauma (11 eyes, 2.73%), a history of endophthalmic surgery (trabeculectomy or scleral buckling; 6 eyes, 1.49%), atopic dermatitis (3 eyes, 0.75%), and pigmentary degeneration of the retina (3 eyes, 0.75%). The study followed the tenets of the Declaration of Helsinki, and all subjects provided written informed consent after receiving an explanation of the nature and possible consequences of participation. The Institutional Review Board of Kozawa Eye Hospital and Diabetes Center approved the study (No. 14).

### Surgical procedure

**Fig 1:**
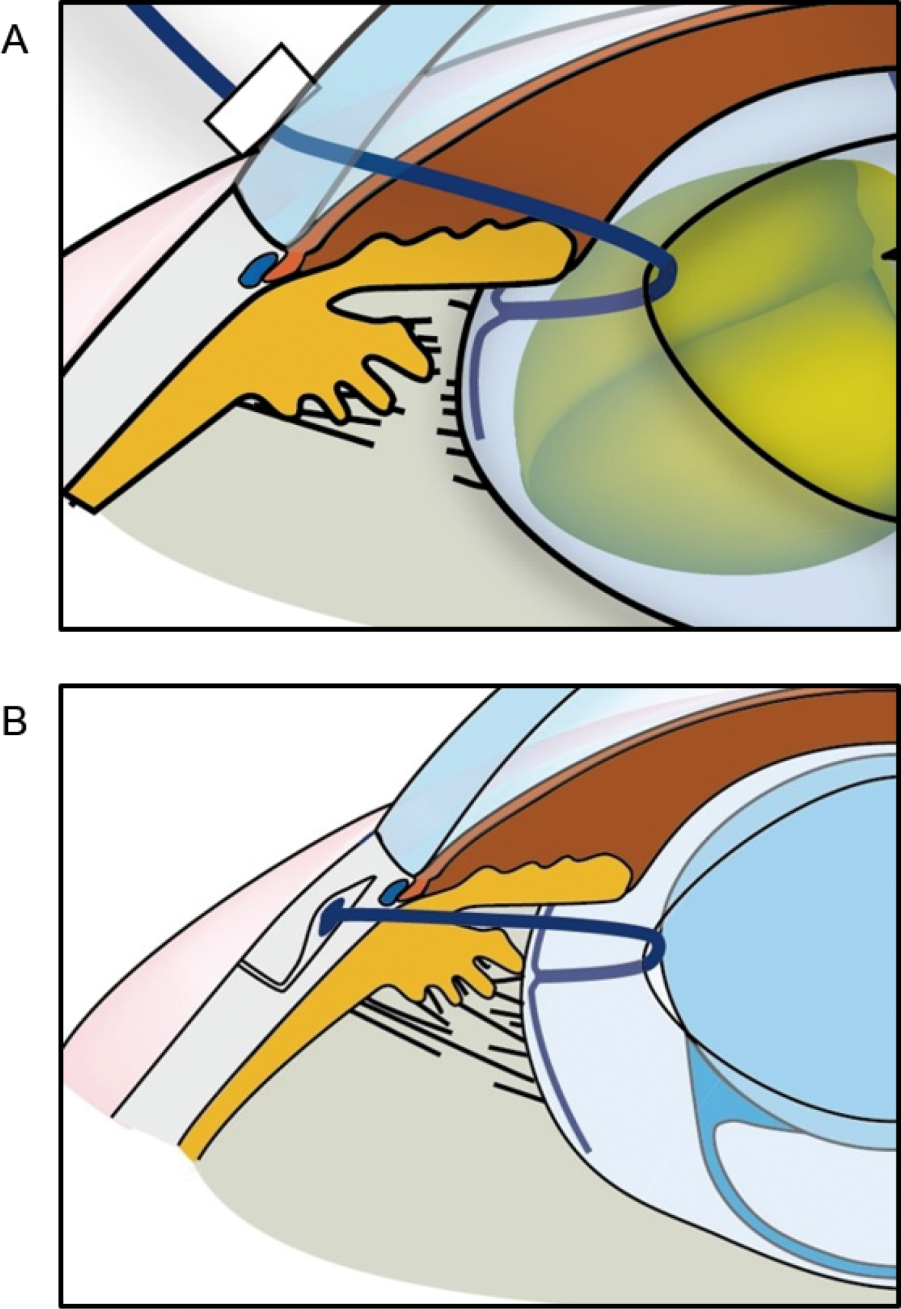
Capsule expander (CE) and modified-capsule expander (M-CE). (A) The CE simultaneously expands the capsular equator and the edge of the continuous curvilinear capsulorhexis by a T-shaped footpad during phacoemulsification and aspiration performed in eyes with weak zonules. (B) The M-CE permanently fixates the lens capsule to the sclera with the intraocular lens inserted in the lens capsule in eyes with extensive or progressive zonular compromise.

All the cataract surgeries were performed by the same surgeon (S.Y.) under topical anesthesia. After creating clear corneal and stab incisions, ophthalmic viscosurgical device (OVD) (Healon®, Abbot Medical Optics Inc., Illinois, USA) was injected into the anterior chamber so that the anterior chamber was completely filled with OVD and until OVD refluxed from the paracentesis site. CCC of the anterior lens capsule was performed using a cystotome (a 27-G needle). The procedure was continued using the needle to puncture the capsule in the periphery, extending the tear, gently pushing the peripheral margin with the needle tip until it was parallel to the edge of the pupil, and then tilting the needle to 45° to properly catch and scratch the capsule. Emulsification of the lens nucleus and aspiration of the lens cortex were performed using the semi-crater and split technique [9] with a PEA machine (INFINITI^®^ Vision System, Alcon, Texas, USA). An acrylic foldable IOL was implanted after the capsular bag was filled with an OVD. If the conventional PEA and in-the-bag IOL fixation could not be performed and completed, an additional use of device and additional surgical procedure were performed. We used a highly retentive and cohesive OVD (Healon5^®^, Abbot Medical Optics Inc., Illinois, USA) and a capsule stabilization device in cases with zonular weakness. When CCC could not be performed because of severe lens movement, Healon5^®^ was injected into the anterior chamber. We used a capsule expander (CE, Handaya Co. Ltd., Tokyo, Japan) [10], an iris retractor, or/and a CTR during PEA. We have developed a CE to preserve the lens capsule integrity during PEA in cases with weak zonules. The contact portion of the CE is bent at 1.25 mm with an end bifurcating to form a 2.0-mm T shape to simultaneously expand the capsular equator and the edge of CCC (Fig 1). If it was determined that the IOL could not be fixed in an unstable capsular bag, a scleral suture fixation of the IOL or in-the-bag IOL fixation following scleral fixation of the lens capsule using a modified capsule expander (M-CE, Handaya) [11] was performed. We have developed an M-CE that permanently fixes the lens capsule to the sclera with the IOL inserted in lens capsules in eyes with extensive or progressive zonular compromise (Fig 1).

### Measurement of the shifted distance of the lens

**Fig 2:**
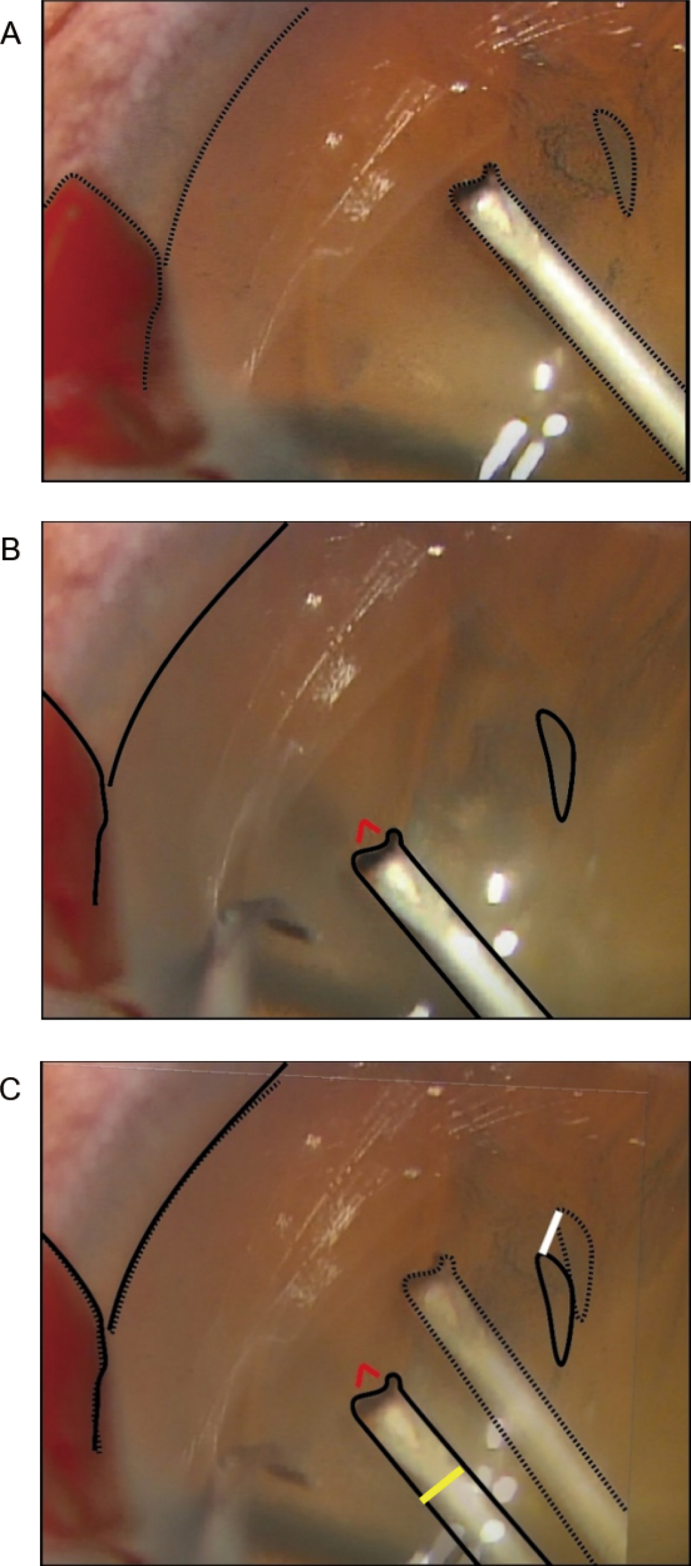
Measurement of the shifted distance of the lens in a patient with cataract. Two images were captured, one showing when the needle tip caught the anterior capsule (A) and the other showing when the needle tip tore the capsule (B; the red line indicates the tear). The margins of the needle, pupil, and cortical opacity were traced, and the two images were overlaid such that the pupillary margins coincided (C). The distance moved by the cortical opacity (white line) was calculated based on the diameter of the cystotome (yellow line).

The distance shifted by the lens was measured using a previously reported method^7^. In brief, a video recording of the CCC procedure was made at high magnification and captured using a film editing software (Windows Live Movie Maker, Microsoft Corporation, Washington, USA) and then played back frame by frame with an interval of 0.033 s. Two images were captured: one when the needle tip caught the anterior capsule and the other when the needle tip tore the capsule. The margins of the needle, pupil, pupil pattern, and cortical opacity in two images were traced using an illustration software (Illustrator CC, Adobe Systems Inc., San Jose, CA, USA) with dotted lines for one image and solid lines for the other (Fig 2). The two images were overlaid to coincide with the pupillary margin and pupil pattern. The shifted distance of the cortical opacity within 3 mm from the cystotome, shown by a misalignment in the image between the dotted and solid lines, was regarded as the shifted distance of the lens. In cases where no distinct cortical opacities were observed, the indentation made on the lens surface by the hook was used as a mark for measuring the shifted distance. The overlaid images were imported into measurement software (Microsoft Excel, Microsoft Corporation, Washington, USA) to measure the shifted distance of the corneal opacities, which was calculated based on the diameter of the cystotome (Fig 2).

### Objective classification of zonular weakness

Zonular weakness was classified into three grades based on the shifted distance of the lens: Grade I, less than 0.20 mm; Grade II, 0.20–0.39 mm; and Grade III, greater than 0.40 mm.

### Statistical analysis

The Mann–Whitney U test was used to examine the correlation between the shifted distance of the lens and the presence of anterior capsule striae. The Spearman’s rank correlation coefficient (r) was used to determine correlations between the subjective and objective classification systems. A p value of <0.05 was considered statistically significant.

## Results

### Shifted distance of the lens

**Fig 3:**
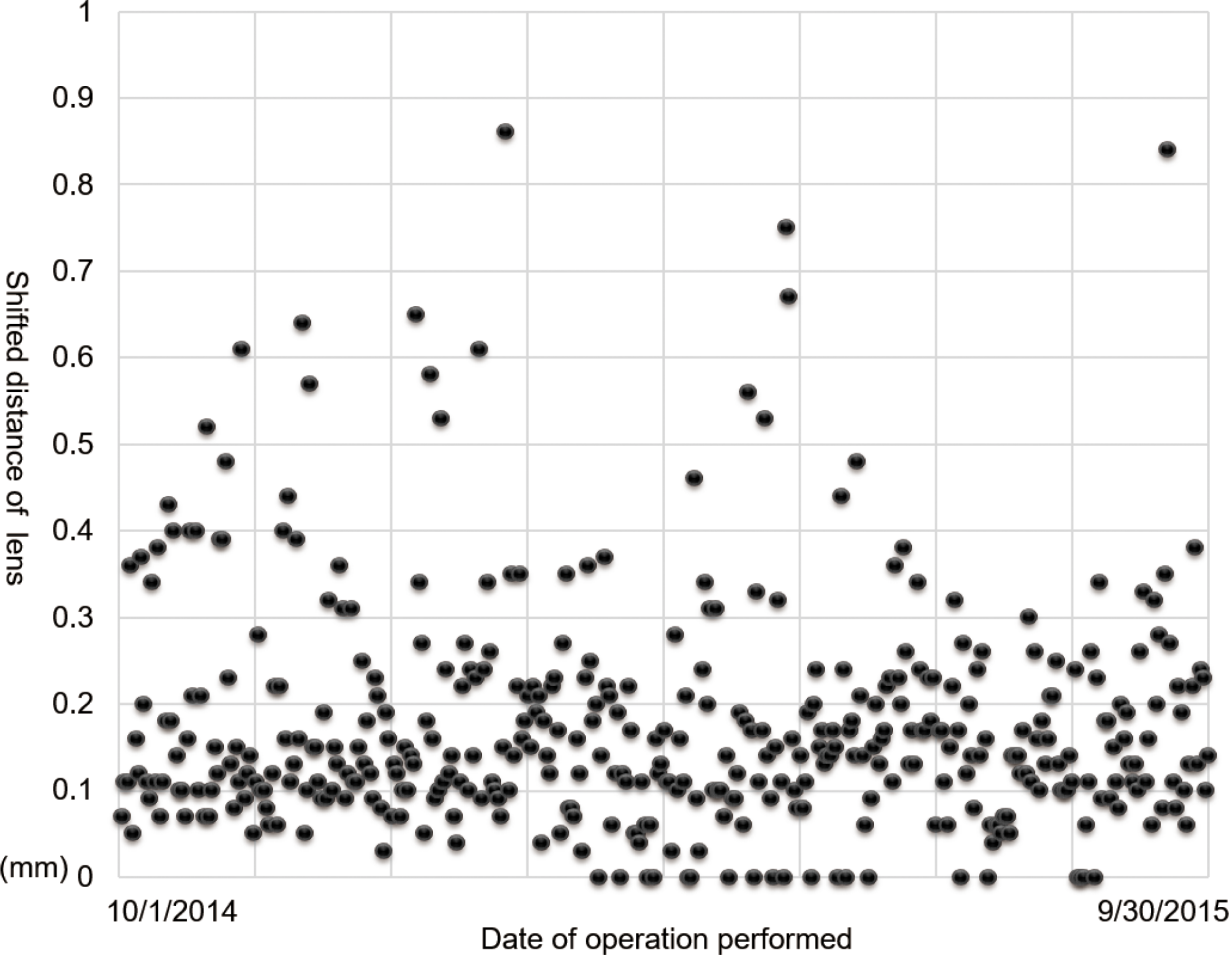
The shifted distance of the lens in all cases (N = 402). The mean shifted distance was 0.18 ± 0.13 mm.

We examined the shifted distance of the lens during cataract surgery. The mean shifted distance was 0.18 ± 0.13 mm (range, 0.00–0.86 mm). The distribution of shifted distance for all the eyes studied is shown in Fig 3.

### Objective classification of zonular weakness

The classification of zonular weakness with respect to the 402 eyes is shown in Table 1. We classified 276 eyes (68.66%) as Grade I (<0.20 mm), 102 eyes (25.37%) as Grade II (0.20–0.39 mm), and 24 eyes (5.97%) as Grade III (≥0.4 mm).

**Table 1:** Classification of zonular weakness.

Grade	N (%)
Grade I (<0.20 mm)	276 (68.66%)
Grade II (0.20–0.39 mm)	102 (25.37%)
Grade III (≥0.4 mm)	24 (5.97%)

### Surgical procedure for lens removal

**Fig 4:**
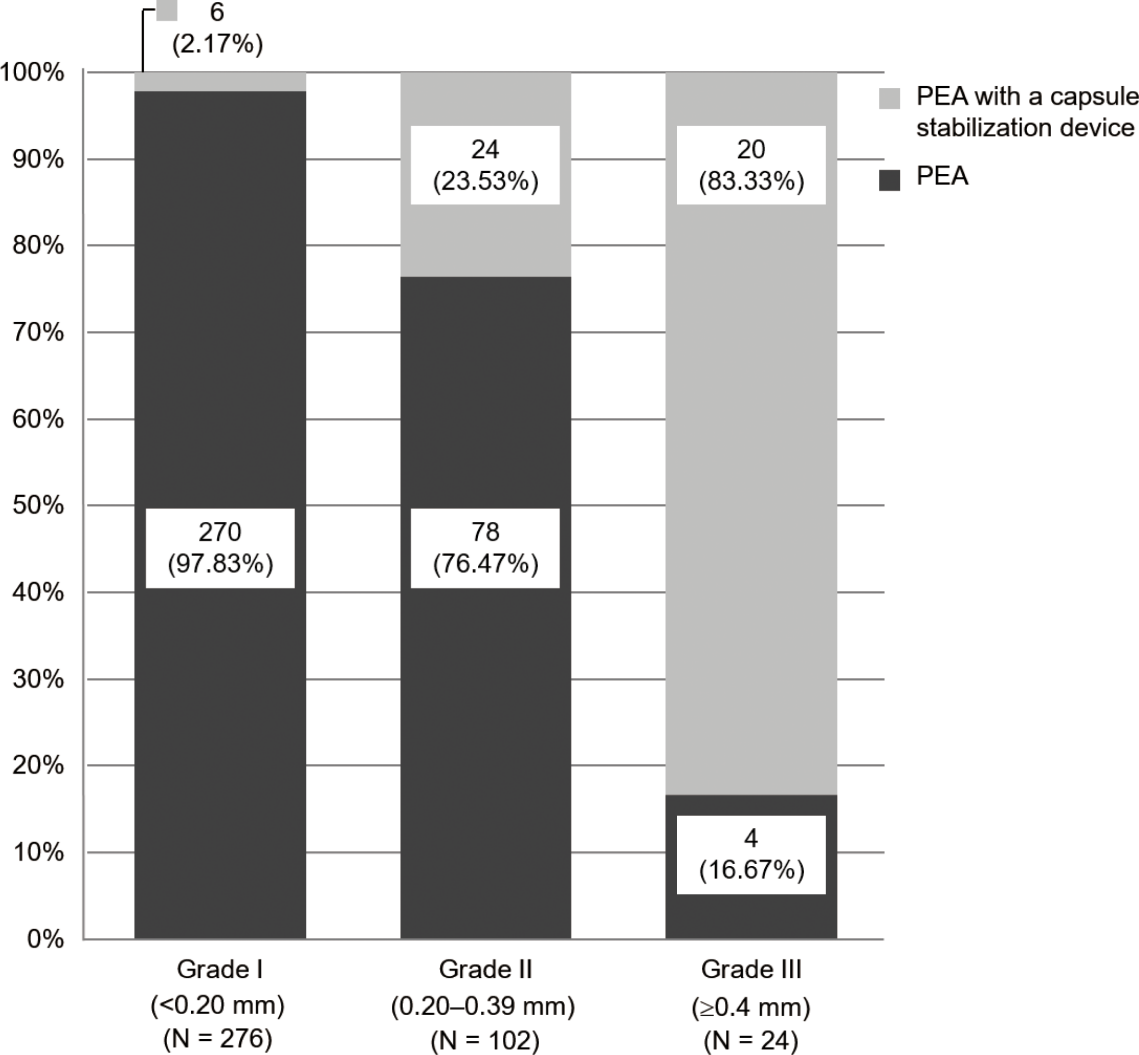
Surgical procedure for lens removal. The use of a capsule stabilization device during phacoemulsification and aspiration increased as the zonular weakness grade (I–III) increased.

Standard PEA or PEA with a capsule stabilization device was performed for lens removal in this study. Standard PEA required a capsule stabilization device when there was zonular dehiscence. The number and percentage of eyes in each grade are shown for each of procedures in Fig 4. The use of a capsule stabilization device during PEA increased at higher grades. Of the 402 eyes examined, 50 (12.4%) required a capsule stabilization device. The types of capsule stabilization devices used are indicated in Table 2.

**Table 2:** Capsule stabilization device usage.

Capsule stabilization device	N (%)
CE	25 (50.0%)
Iris retractor	12 (24.0%)
CTR	4 (8.0%)
CTR in combination with CE	8 (16.0%)
CTR in combination with iris retractor	1 (2.0%)

CE, capsule expander; CTR, capsular tension ring

### Surgical procedure for IOL fixation

**Fig 5:**
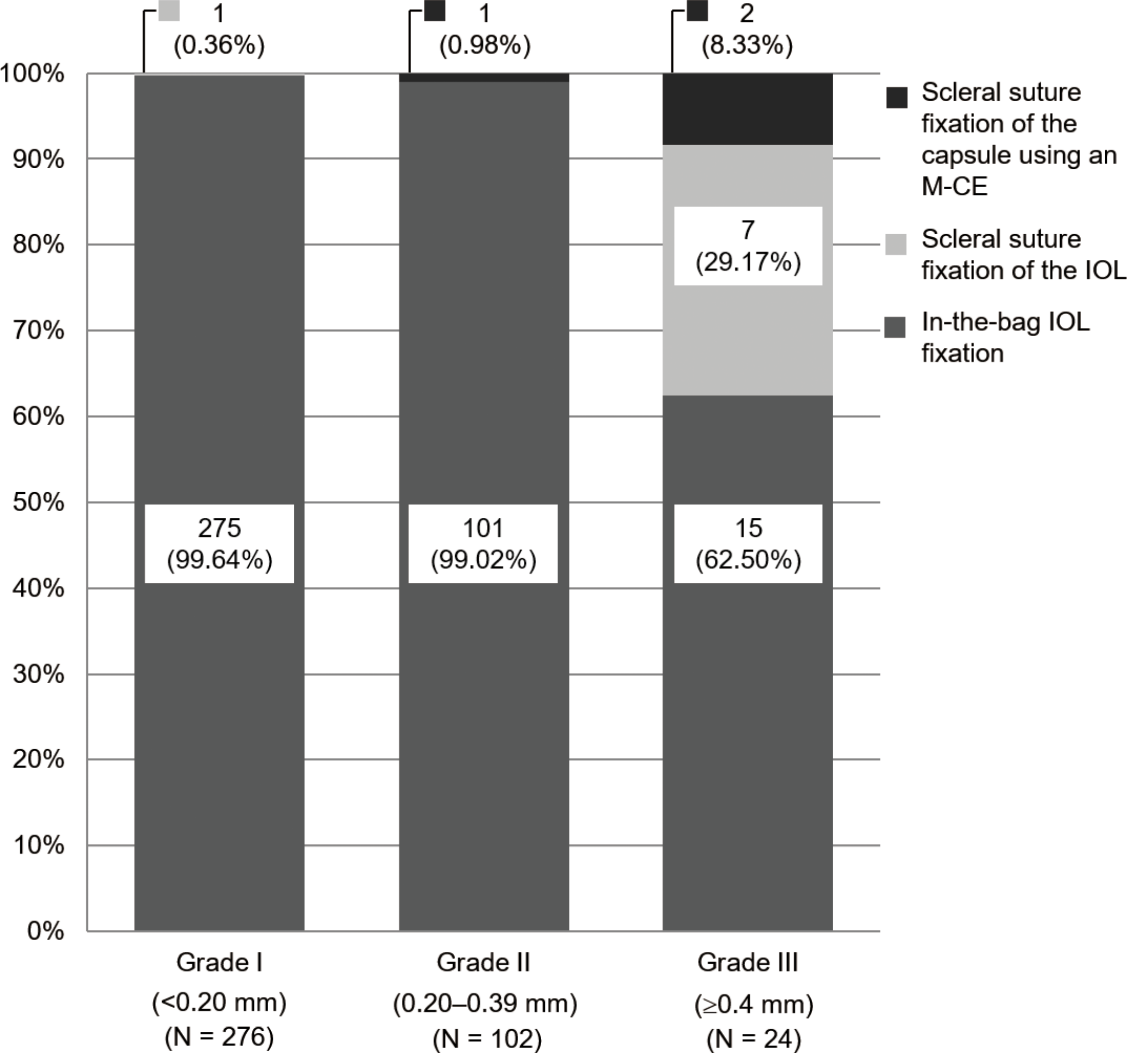
Surgical procedure for intraocular lens (IOL) fixation. The rate of scleral suture fixation of the IOL and scleral suture fixation of the capsule using a modified-capsule expander increased as the zonular weakness grade (I–III) increased.

IOL fixation included in-the-bag IOL fixation, scleral suture fixation of the IOL, and in-the-bag IOL fixation following scleral fixation of the lens capsule using a M-CE [11]. Fig 5 shows the number of each type of IOL fixation procedure used for each zonular weakness grade. The use of scleral suture fixation of the IOL and scleral suture fixation of the capsule using an M-CE increased as the grade increased. Scleral suture fixation of the IOL was performed in one Grade I eye (0.36%) and seven Grade III eyes (29.17%). Scleral suture fixation of the capsule using an M-CE was performed in one Grade II eye (0.98%) and two Grade III eyes (8.33%). The IOLs remained centered and stable following the procedure.

### Anterior capsule striae

**Fig 6:**
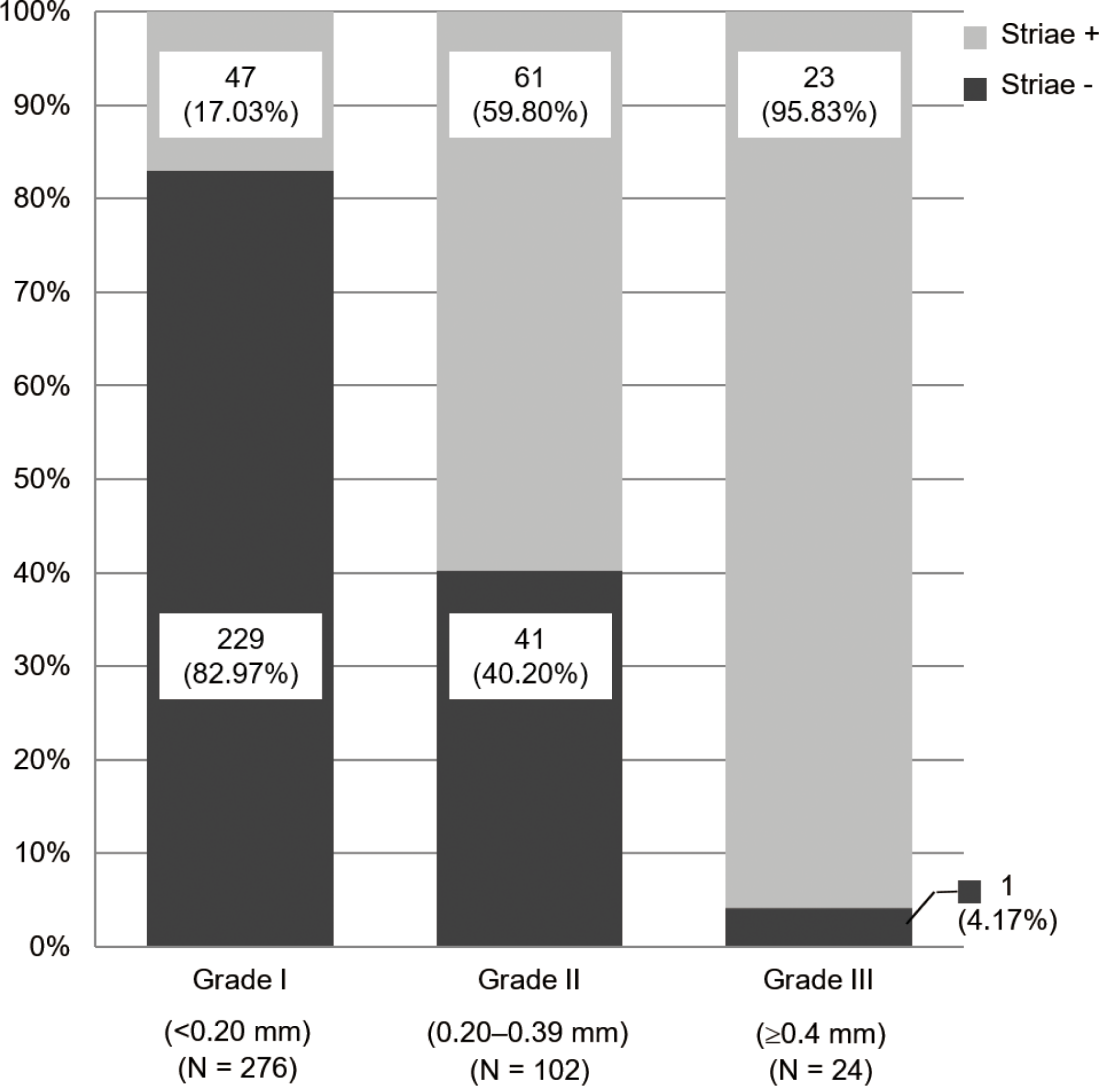
The presence of anterior striae according to zonular weakness grade. There was a statistically significant correlation between the shifted distance of the lens and the presence of anterior capsule striae. (Mann–Whitney U test, p < 0.001)

In addition to the movement of the entire capsular bag during CCC, anterior capsule striae during CCC comprise a characteristic sign of zonular instability [6]. We examined the correlation between the lens movement and presence of the anterior capsule striae during CCC to verify the validity of the classification system. Fig 6 shows the proportion of eyes in which there were anterior striae in each zonular weakness grade. There was a statistically significant correlation between the shifted distance of the lens and presence of anterior capsule striae (p < 0.001).

## Discussion

The ciliary zonules consist mostly of a series of fibers passing from the ciliary body to the lens. They hold the lens in position and enable the ciliary muscle to act during accommodation [12]. Zonule fibers consist of microfibrils with a tubular structure that are arranged in bunches of filaments. They possess characteristics similar to elastin fibers and are highly elastic [13]. Weakness of the zonule can be caused by various factors, including aging [12], PXS [14], connective tissue disorders such as Marfan syndrome [15], retinitis pigmentosa [16], post-laser iriodotomy [17], atopic dermatitis [18], iatrogenic zonular dehiscence induced during vitreoretinal surgery [19], and complex cataract surgery, as well as external injury [20]. It has become important for cataract surgeons to have the ability to identify zonular weakness and manage patients with weak zonules.

A complete CCC is paramount for successful PEA and bag preservation in cases with weak zonules. In normal cases where the zonules are intact, CCC can be performed without difficulty and is accompanied by no or only slight lens movement. However, in cases with weak zonules, CCC is more challenging because special consideration is required for its initiation and completion. Puncturing the anterior capsule is difficult because zonular counter traction is compromised [21]. Zonular strength can best be assessed through direct maneuvers; findings at the initiation and during CCC can be used to make a judgment of the degree of zonular instability [6, 7]. We have previously reported a classification for zonular weakness based on findings at CCC and the difficulty in performing CCC [22]. The classification definitions were as follows: normal, with no or slight lens movement at the start of CCC and no difficulty in performing CCC; weak, with moderate lens movement during CCC and some difficulty in puncturing the anterior capsule and extending the tear; very weak, due to severe lens movement and difficulties with the initial puncture, CCC can be performed with the occasional aid of Healon5^®^; and extremely weak, with zonular deficiency in addition to the very weak criteria and accounted for the cases of severe phacodonesis, lens subluxation, lens luxation into the anterior chamber, and dropped nucleus into the vitreous cavity that could be classified preoperatively. We examined the surgical procedure for lens removal and IOL fixation for each of these classifications and found that the use of capsular stabilization devices during PEA, the rate of ICCE, and scleral suture fixation of the IOL all increased as the weakness classification increased. We concluded that classifying zonular weakness was useful for selecting an appropriate capsule stabilization device and procedure during cataract surgery. The classification system was based on 5,447 interventional cases where all the cataract surgery was performed by a single experienced surgeon [22]. This has the advantage that the classification was based on findings from a single surgeon who performed the same CCC procedure, a disadvantage is that it is based on the subjective findings of that single surgeon.

To solve this problem, in the present study, we aimed to develop a new classification system based on objective findings. Zonular weakness is often described according to the number of clock hours, which reflect the degree of zonular dialysis [21, 23, 24]. In the present study, we quantified zonular weakness by measuring the shifted distance of the lens at the start of CCC and created an objective classification system based on this shift. We investigated the surgical procedures used with each zonular weakness grade. It has been reported that grading the severity of zonulopathy is useful, as the choice of cataract extraction and a capsule stabilization device depends greatly on the degree of zonular instability [21]. When our objective classification of weak zonules was applied, we found the need for a capsule stabilization device during PEA and scleral suture fixation of the IOL increased with greater zonular weakness grade. These results suggest the usefulness of this classification system for selecting an appropriate capsule stabilization device and procedure during cataract surgery. Furthermore, we demonstrated a correlation between lens movement and the presence of anterior capsule striae during CCC. Anterior capsule striae are observed in the presence of zonular instability at the start of CCC because of undue tension on the weak zonule [6]. As lens movement and anterior capsule striae are two major signs of zonular instability observed during CCC [6], the positive correlation between degree of lens movement and presence of striae suggests the validity of this classification system.

There were limitations to our study. First, the surgical techniques applied in CCC, such as the instruments used (cystotome or forceps), the access and approach taken (via a primary incision or a side-port paracentesis), the medium used (balanced salt solution or OVD), and the amount of OVD used depend on the surgeon. The shifted distance of the lens would be affected by these factors. The findings associated with CCC need to be examined under different conditions, and the applicability of the classification to other cataract surgeons performing CCC with their own techniques should also be considered. Second, we have to accommodate the new objective classification system with our previous subjective classification system. We compared the distribution of the three zonular weakness grades with our previous study’s subjective classification system (normal, weak, and very weak, described earlier); the results are shown in S1 Fig. A positive correlation was observed between the two systems (r = 0.82, p < 0.001). If a clear distinction can be made between groups of classifications of zonular weakness, it may become clearer and easier to understand. Third, the classification system of the present study is based on lens movement during CCC. Although findings at the initiation and during CCC are considered to be the litmus test of zonular instability, these provide a limited indication under limited conditions. Other intraoperative signs of zonular instability include posterior capsule laxity, striae during cortex removal, the collapse of the lens equator, and vitreous prolapse peripherally around the capsule bag [6]. We plan to examine the relationship between the presence of these signs and the degree of zonular instability. Furthermore, there are cases that need special consideration in determining the zonular weakness, such as age-related changes of the zonules and PXS. Zonules become more fragile with age [25]. Fetal and infant zonular fibers are finer and less aggregated than those in adults. Zonules decrease in number and the fibers become finer and sparser and rupture more readily in the elderly [11]. We sometimes encounter progressive zonular dialysis throughout cataract surgery in elderly patients. The potential strength of the zonule would differ depending on the patient’s age, which is something that cannot be assessed during CCC. PXS causes progressive zonular weakness, which results in capsule contraction syndrome and in-the-bag IOL dislocation postoperatively [7, 26]. We cannot predict these risks intraoperatively. We plan to examine the relationship between the shifted distance and the risks of capsule contraction syndrome and in-the-bag IOL dislocation. Fourth, measurement methods that can more accurately reflect zonular weakness should be examined. The pressure to puncture the anterior capsule at the initiation of CCC may not be strong enough to reflect zonular strength. Zonular instability could be assessed more accurately in situations where a greater direct load is applied to the lens capsule, such as PEA. We plan to examine the shifted distance of the lens capsule throughout PEA. Fifth, this classification cannot be utilized during real time surgery unless eyes were classified immediately. Image guided system makes premium IOL surgery precise, fast and easy. It directly project the assistance functions of corneal incision, capsulorhexis, and toric IOL alignment in the surgical field. We think it would be very useful if the lens movement at the start of CCC can be measured intraoperatively, and the classification appear in the surgical field. Furthermore, if the alarm sign appear in cases with long shifted distance, defined as Grade III, surgeon can be aware of the zonular weakness immediately.

In conclusion, we report an objective classification of weak zonules determined by the shifted distance of the lens during CCC in patients undergoing cataract surgery. The use of a capsule stabilization device and scleral suture fixation of IOL increased with greater zonular weakness grade. This classification of zonular weakness could be useful for selecting an appropriate capsule stabilization device and procedure during cataract surgery.

## Supporting information

S1 Fig Application of the previous subjective zonular classification system of normal, weak, and very weak to each grade of the objective zonular weakness classification system. A positive correlation was found between the two classification systems (r = 0.82, p < 0.001, Spearman’s rank-correlation coefficient).
(TIF)
