# Supplementary figures and images for: Objective classification of zonular weakness based on lens movement at the start of capsulorhexis
Source: PLoS One. 2017 Apr 20;12(4):e0176169. doi: 10.1371/journal.pone.0176169 (PMC5398681; doi:10.1371/journal.pone.0176169)

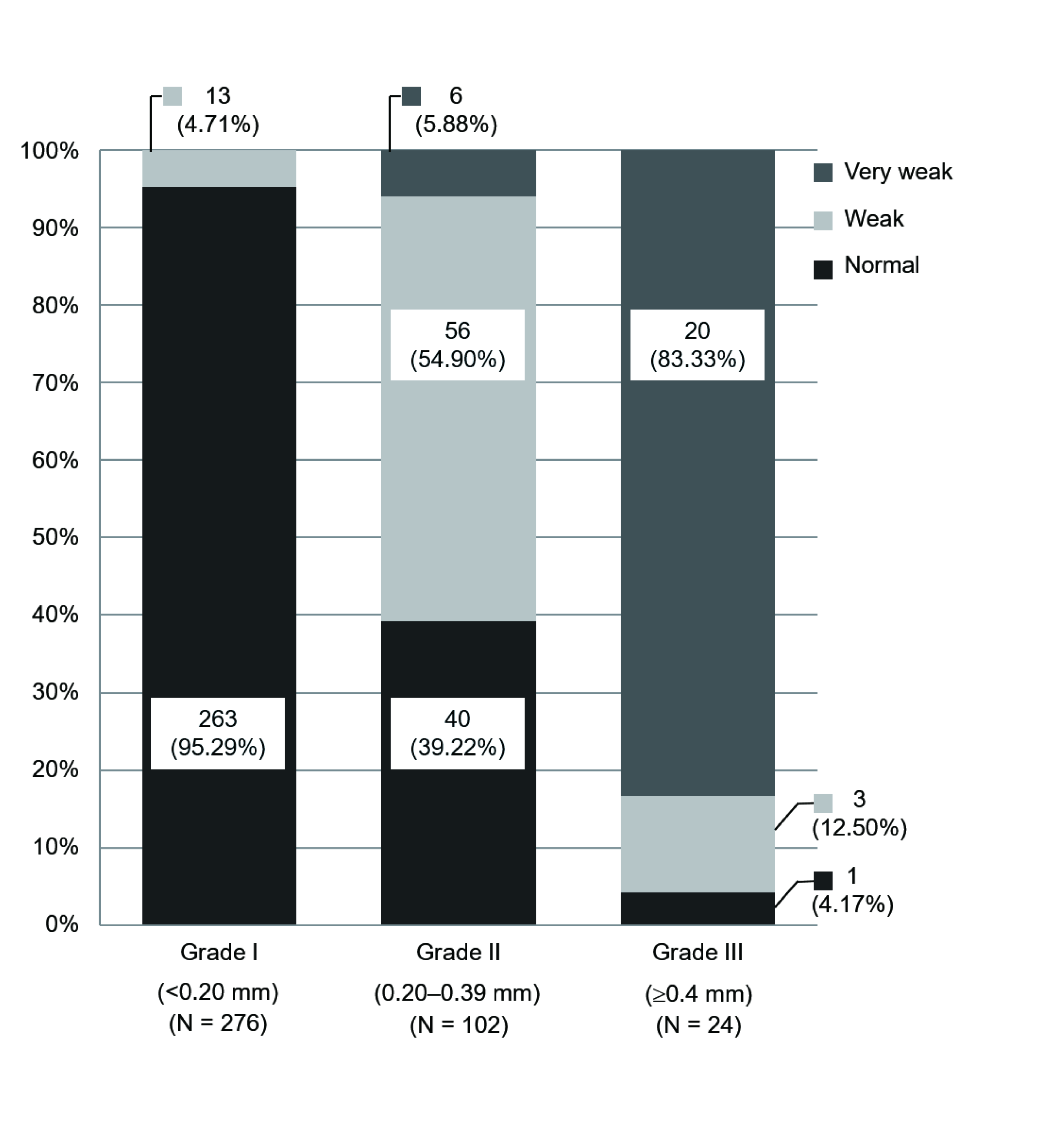

Supplement: S1 Fig — A positive correlation was found between the two classification systems (r = 0.82, p < 0.001, Spearman’s rank-correlation coefficient). (TIF) [file pone.0176169.s001.tif]
